# Lipidomic changes in the liver of beagle dogs associated with *Toxocara canis* infection

**DOI:** 10.3389/fcimb.2022.890589

**Published:** 2022-09-13

**Authors:** Hao-Yu Li, Yang Zou, Hany M. Elsheikha, Yue Xu, Lang Cai, Shi-Chen Xie, Xing-Quan Zhu, Wen-Bin Zheng

**Affiliations:** ^1^Laboratory of Parasitic Diseases, College of Veterinary Medicine, Shanxi Agricultural University, Taigu, China; ^2^State Key Laboratory of Veterinary Etiological Biology, Key Laboratory of Veterinary Parasitology of Gansu Province, Lanzhou Veterinary Research Institute, Chinese Academy of Agricultural Sciences, Lanzhou, China; ^3^Faculty of Medicine and Health Sciences, School of Veterinary Medicine and Science, University of Nottingham, Loughborough, United Kingdom; ^4^Key Laboratory of Veterinary Public Health of Higher Education of Yunnan Province, College of Veterinary Medicine, Yunnan Agricultural University, Kunming, China

**Keywords:** *Toxocara canis*, toxocariasis, beagle dog, liver, lipidomics

## Abstract

A global lipidomic analysis using liquid chromatography–tandem mass spectrometry was performed on the liver of beagle dogs infected with *Toxocara canis* to profile hepatic lipid species at 12 h post-infection (hpi), 24 hpi, and 36 days post-infection (dpi). This analysis identified six categories and 42 subclasses of lipids, including 173, 64, and 116 differentially abundant lipid species at 12 hpi, 24 hpi, and 36 dpi, respectively. Many of the identified lysophospholipids, such as lysophosphatidylglycerol, lysophosphatidylserine, and lysophosphatidylcholine, may contribute to the migration and development of *T. canis* during the early infection stage. Pathway analysis revealed significant alterations of several immune-inflammatory pathways, such as the B-cell receptor signaling pathway, the NF-kappa B signaling pathway, and the C-type lectin receptor signaling pathway at 12 and 24 hpi. These findings demonstrate the value of lipidomic profiling in revealing the extent of changes in the composition and abundance of hepatic lipidome caused by *T. canis* infection and their relevance to the pathophysiology of toxocariasis in beagle dogs.

## Introduction

Toxocariasis, caused by the cosmopolitan helminths of the genus *Toxocara*, is a neglected parasitic zoonotic disease (Ma et al., 2018), especially in the tropical and subtropical regions, as well as in disadvantaged communities (Holland, 2017). *T. canis* eggs are passed in the feces of the canid definitive host, causing extensive environmental contamination (Overgaauw and van Knapen, 2013; Fakhri et al., 2018). Humans become infected *via* accidental ingestion of food or water contaminated by embryonated *T. canis* eggs, which can lead to visceral larva migrans (VLM), ocular larva migrans (OLM), or neurotoxocariasis (NT) (Chen et al., 2018).

There is a growing demand for a more comprehensive understanding of how *T. canis* interacts with and evades the host immune responses (Despommier, 2003; Maizels, 2013; da Silva et al., 2018). It is noteworthy that many non-protein molecules with immunomodulatory properties have been identified in *T. canis* excretory–secretory products (ESPs) (Wangchuk et al., 2020). Global serum metabolomic profiling has also shown that *T. canis* infection can alter lipid signaling pathways, such as biosynthesis of the unsaturated fatty acids pathway and biosynthesis of the steroid hormone pathway (e.g., progesterone and estradiol) in beagle dogs (Zheng et al., 2019), suggesting that lipids may play important roles in *T. canis* infection. The application of high-throughput omics (e.g., genomics, transcriptomics, and proteomics) technologies has proven to be extremely valuable in providing novel insights into the pathophysiology of toxocariasis (Zhu et al., 2015; Zheng et al., 2020). However, there are no studies available that characterize the liver lipidomic profile and discuss its relevance to the pathogenesis of toxocariasis in dogs.

Lipids are essential metabolites, which contribute to various metabolic, immunologic, and signaling processes in cells and organisms (Muro et al., 2014; Kawai et al., 2021). According to the LIPID MAPS database (https://www.lipidmaps.org/), lipids are divided into eight categories, namely fatty acyls (FA), glycerolipids (GL), glycerophospholipids (GP), polyketides (PK), prenol lipids (PR), saccharolipids (SL), sphingolipids (SP), and sterol lipids (ST). Recent advances in coupling liquid chromatography with tandem mass spectrometry have enabled the comprehensive identification and quantification of a plethora of lipid species in biological tissues and fluids, providing a powerful means for the identification of biomarkers to improve risk prediction of the disease (Meikle et al., 2021; Todorović et al., 2021).

*T. canis* has a complex life cycle that involves larval migration to the liver at 12–24 h post-infection (hpi) (Schnieder et al., 2011; Bowman, 2020). In this study, we tested the hypothesis that lipidomics can assist in identifying compositional differences in the lipid species between dogs infected by *T. canis* and uninfected dogs. Using a global lipidomic approach, we monitored the temporal changes of lipid metabolites in the liver of beagle dogs infected by *T. canis* using liquid chromatography–tandem mass spectrometry (LC-MS/MS). Our data identified many lipid species that mediate the interaction between *T. canis* and liver of the canid host, providing more insight into the pathophysiology of toxocariasis.

## Materials and methods

### Animal infection and collection of liver samples

Six- to seven-week-old, specific pathogen free (SPF) beagles dogs used in this study are from the same cohort used in a previous study (Zou et al., 2020). Forty-four puppies of both genders were divided into three groups according to the stage of *T. canis* development in the canine host: 12 h group (*n* = 16, 7 infected vs. 9 control), 24 h group (*n* = 13, 7 infected vs. 6 control), and 36 d group (*n* = 15, 7 infected vs. 8 control). Puppies from the same litters were randomly allocated to infected groups (I) and control groups (C). Puppies in the infected groups were orally inoculated with 300 embryonated *T. canis* eggs in 1 mL normal saline, whereas puppies in the control groups were inoculated with an equal amount of normal saline only. The liver samples were obtained from the same anatomical site of all puppies at 12 hpi, 24 hpi, and 36 days post-infection (dpi) as previously described (Zheng et al., 2019) and stored at –80°C.

### Sample preparation and lipid extraction

Twenty-five milligrams of each liver sample was thawed at 4°C and placed in 1.5 mL sterile tube containing two small steel balls. Then, 800 μL dichloromethane and methanol (v: v = 3:1, –20°C) and 10 μL of SPLASH internal standards solution (330707, Avanti Polar Lipids, USA) were added into the liver sample, followed by grinding using a tissue grinder (50 Hz, 5 min). The ground liver sample was further processed by ultrasonic treatment in water bath at 4°C for 10 min and cooled at –20°C for 1 h. Subsequently, 600 μL of the supernatant was lyophilized after centrifugation at 25,000 rpm for 10 min at 4°C, and reconstituted with 200 μL of isopropanol, acetonitrile, and water (v:v:v = 2:1:1). The sample was shaken for 1 min, followed by ultrasonic treatment and centrifuged as above. Then, the supernatant was transferred to a 1.5 mL vial. Quality control (QC) samples were prepared by pooling 20 μL of each sample to evaluate the stability and repeatability of LC-MS/MS analysis. The separation and detection of lipids in each liver sample and QC sample were performed by using Waters 2D UPLC (Waters, USA) and a Q-Exactive high-resolution mass spectrometer (Thermo Fisher Scientific, USA).

### The UPLC-MS/MS analysis

A CSH C18 column (1.7 μm 2.1*100 mm, Waters, USA) was used in this study. Under the positive ion mode (ESI^+^), the mobile phase included solvent A [60% acetonitrile aqueous solution (ACN), 0.1% formic acid (FA), and 10 mM ammonium formate (AF)] and solvent B (10% ACN, 90% isopropanol, 0.1% FA, and 10 mM AF). Under the negative ion mode (ESI^−^), the mobile phase comprised of solvent A (60% ACN and 10 mM AF) and solvent B (10% ACN, 90% isopropanol, and 10 mM AF). Gradient elution conditions were set as follows: 40% to 43% mobile phase B for 0–2 min, 43% to 50% mobile phase B for 2–2.1 min, 50% to 54% mobile phase B for 2.1–7 min, 54% to 70% mobile phase B for 7–7.1 min, 70% to 99% mobile phase B for 7.1–13 min, 99% to 40% mobile phase B for 13–13.1 min, and 40% mobile phase B for 13.1–15 min with a constant flow of 0.35 μL/min at 55°C. The lipids separated by liquid phase were injected into the Q-Exactive mass spectrometer to obtain MS1 and MS2 data with the range of 200–2,000 m/z. In the MS1 analysis, one full MS scan was conducted (resolution: 70,000; maximum injection time (MIT): 100 ms; automatic gain control (AGC): 3E6). According to the precursor ion intensity, the top 3 ions were selected for subsequent MS2 analysis (resolution: 17,500; MIT: 50 ms; AGC: 1E5), and collision energy (stepped a normalized collisional energy) was set as 15, 30, and 45 eV.

### Statistical analysis

LC-MS/MS data analysis was performed by LipidSearch v.4.1 (Thermo Fisher Scientific, USA), including intelligent peak extraction, lipid identification and peak alignment, and subsequent data analysis. Multivariate and univariate analyses were conducted using the metaX R package, including deletion of the lipid species missing more than 50% of QC samples and more than 80% of experimental samples, normalization of LC-MS/MS data, and deletion of the lipid species whose CV (coefficient of variation) of the relative peak area was > 30% in all QC samples (Wen et al., 2017). The quality of LC-MS/MS data was examined using QC samples, including chromatogram overlap of QC samples, PCA (principal component analysis), peak number, and peak response intensity difference. The PCA and PLS-DA (partial least squares method—discriminant analysis) were used to perform the multivariate statistical analysis. Furthermore, quantitative analysis of lipid subclasses was performed to reveal the relative amounts of lipid subclasses in each infected and control group. The lipids that met the following conditions were considered as differentially abundant lipids: VIP ≥ 1, fold change ≥ 1.2 or ≤ 0.8333, and *p* < 0.05. Hierarchical clustering analysis was performed for differential lipids, and the Euclidian distance was used for distance calculation and Z-score (zero-mean normalization) for data normalization. Metabolic pathway analysis based on the Kyoto Encyclopedia of Genes and Genomes (KEGG; http://www.genome.jp/kegg) database was performed to explore the possible interrelatedness among altered lipid metabolites associated with *T. canis* infection. The conversion of name between the lipid compounds acquired by LC-MS/MS and the KEGG ID in the KEGG database was performed by MetaboAnalyst v.5.0 prior to KEGG enrichment analysis (Pang et al., 2021) (https://www.metaboanalyst.ca/MetaboAnalyst/upload/ConvertView.xhtml). Because multiple lipids with a specific function may correspond to the same KEGG ID, the conversion results were manually selected to match the correct KEGG ID and examine the effect of altered lipids on the metabolic pathways.

## Results

### Global changes of hepatic lipids

The base peak chromatograms overlapping, with little fluctuation of retention time and peak response intensity, showed the stability of the signal during detection and analysis of QC samples (**Supplementary Figure 1**). A total of 1,313 lipid species were identified in QC samples. By calculating the relative standard deviation (RSD) of QC samples, we found that the RSD value of 87.5% of the identified lipids was <20%, and all QC samples were clustered closely by PCA (**Supplementary Figure 2**). Total ion chromatography data showed minor changes in the relative lipid abundances between the infected group and the control group at 12 hpi, 24 hpi, and 36 dpi in both the positive ion mode (ESI^+^) and the negative ion mode (ESI^−^) (**Supplementary Figure 3**). After filtering low-quality ions that had RSD > 30% in QC samples, 1,199 lipids were retained in each sample with 558 and 641 ions identified in ESI^+^ and ESI^−^, respectively, and the RSD ratio was 91.3%, including 938 lipid species (**Supplementary Table 1**). Lipid metabolome focuses on lipid species in a biological sample and thus identifies fewer metabolites compared with full-spectrum metabolome. Therefore, positive and negative ion electrospray ionization modes were combined to obtain a wide range of lipids and comprehensively analyze KEGG pathways of the differentially abundant lipids.

**Figure 1:**
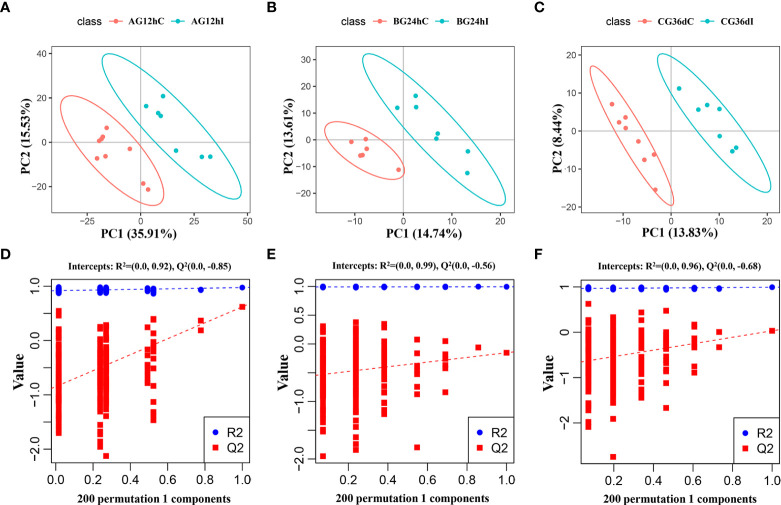
The partial least-squares discriminant analysis (PLS−DA) score scatter plots of lipids **(A–C)** and the response sequencing verification diagram of the PLS-DA model with 200 permutation tests **(D–F)** between the infected groups (I) and control groups **(C)** at 12 hpi, 24 hpi, and 36 dpi, respectively. AG, A group; BG, B group; CG, C group.

The PCA score plots of each group, as shown in **Supplementary Figure 4**, indicate that infected samples and control samples were not well separated at 12 hpi, 24 hpi, or 36 dpi. In contrast, the PLS-DA revealed a clear distinction between infected and control groups between the three infection stages with good fitting and predictive performances (**Figures 1A-C**). To test the predictability of the PLS-DA model, 200 permutation tests were performed, and the calculated R^2^ and Q^2^ values were (0.92 and –0.85), (0.99 and –0.56), and (0.96 and –0.68) at 12 hpi, 24 hpi, or 36 dpi, respectively (**Figures 1D–F**). These results revealed that lipids in infected groups were different from those in the control groups at 12 hpi, 24 hpi, and 36 dpi.

### Temporal lipidomic changes

**Figure 2:**
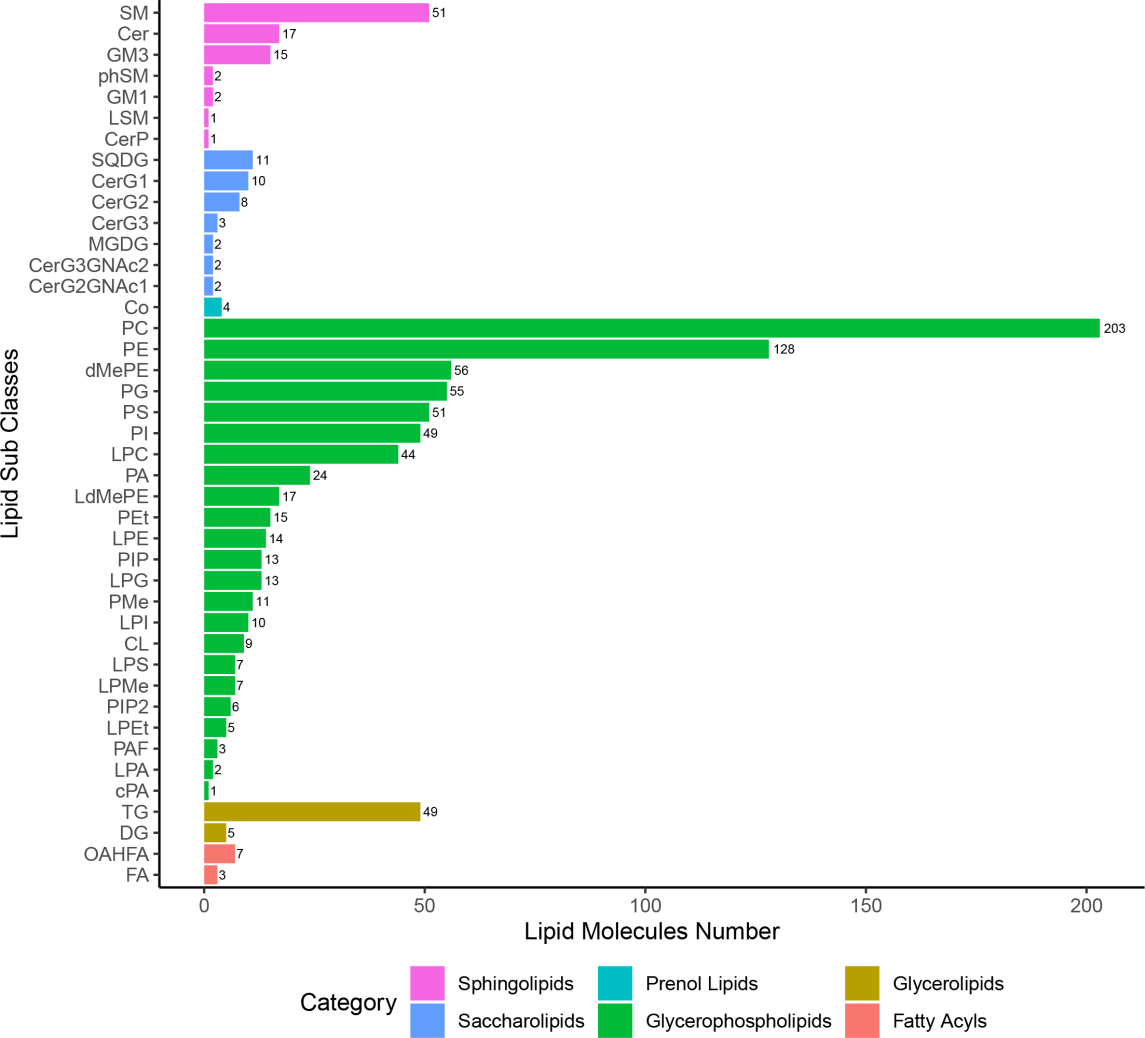
The lipid subclasses/categories and the corresponding number of lipid species per category.

A total of 42 lipid subclasses from six categories were identified, including FA, GL, GP, PR, SL, and SP, but not ST or PK lipid species. Of those, GP was the largest category with 743 lipid species, and phosphatidylcholine (PC) was the largest subclass with 203 lipid species (**Figure 2**). Out of the 42 lipid subclasses, 10, 2, and 4 lipid subclasses were significantly altered at 12 hpi, 12 hpi/24 hpi, and 36 dpi, respectively, which belong to categories GL, GP, SL, or SP (**Supplementary Figure 5**). 47.6% (10/21) lipid subclasses in the GP category were significantly altered at 12 hpi or 36 dpi, including lysophosphatidylglycerol (LPG), lysophosphatidic acid (LPA), and phosphatidylinositol 4,5-bisphosphate (PIP2). Ceramides (Cer) and triglyceride (TG) were significantly altered at 12 and 24 hpi, and sphingomyelin (SM), phosphatidylmethanol (PMe), phosphatidylserine (PS), and CerG3GNAc2 were significantly altered at 36 dpi.

**Figure 3:**
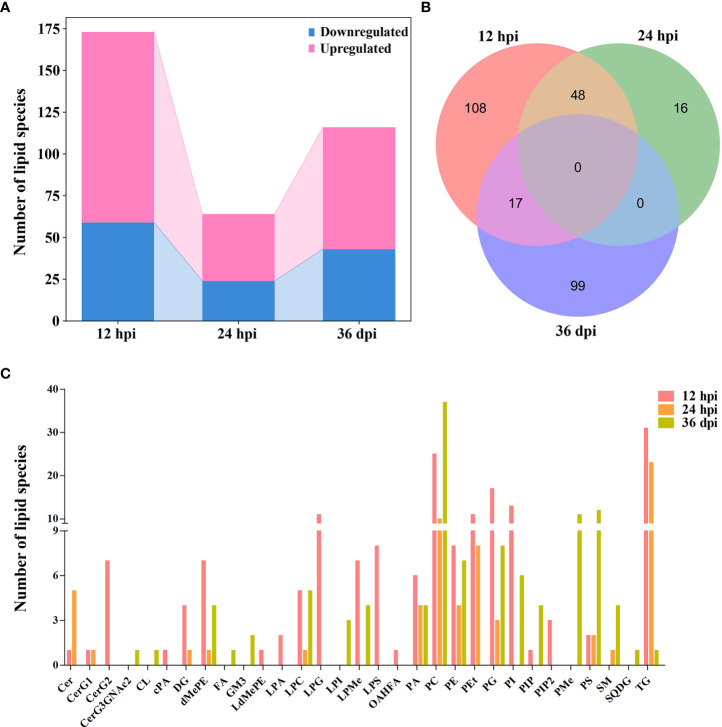
Comparison of lipids with differential abundance in the liver of beagle dogs infected with 300 embryonated *Toxocara canis* eggs at 12 hpi, 24 hpi, and 36 dpi. **(A)** Stacked bar chart shows the numbers of lipids with differential abundance at the three infection stages. **(B)** Venn diagram shows the common and unique differential lipid species among the three infection stages. **(C)** The number of lipid species with differential abundance in each lipid subclass at 12 hpi, 24 hpi, and 36 dpi.

According to the criteria of VIP ≥ 1, FC ≥ 1.2 or ≤ 0.83, and *p* < 0.05, 173, 64, and 116 lipid species showed significant differences between infected and control samples, and the number of upregulated lipid species was markedly greater than that of the downregulated lipid species at the three infection stages (**Supplementary Table 2** and **Figure 3A**). At 12 hpi, 114 lipid species were upregulated and 59 lipid species were downregulated, which belong to 23 lipid subclasses, such as diglyceride [16:0/18:1, DG (16:0/18:1)], phosphatidylethanolamine [18:0/16:0, PE (18:0/16:0)], and lysophosphatidylcholine [22:5, LPC (22:5)]. At 24 hpi, 40 lipid species were upregulated and 24 lipid species were downregulated, which belong to 13 lipid subclasses, such as Cer (d18:1/16:0), DG (16:0/18:1), and PE (22:6/22:6). At 36 dpi, 73 lipid species were upregulated and 43 lipid species were downregulated, which belong to 19 lipid subclasses, such as PC (14:0/14:0), TG (18:1/18:2/20:4), and PS (18:0/18:1). However, there were not any lipid species commonly identified between the three infection stages (**Figure 3B**). The Venn diagrams show that 48 common differential lipid species were identified at 12 and 24 hpi, and the upregulated 34 lipid species and downregulated 14 lipid species were identical in these two infection stages with different abundances, suggesting that these lipid species may play important roles during early stages of *T. canis* infection. There was no identical lipid species with differential abundance between the 24 hpi and 36 dpi groups. Moreover, 17 common lipid species with differential abundances were identified at 12 hpi and 36 dpi, and the upregulated 11 lipid species and downregulated 6 lipid species were identical in these two infection stages with different abundances.

The number of lipid species with differential abundance was counted in each lipid subclass, which showed that some lipid species of diglycosylceramide (CerG2, 7 lipid species), LPG (11 lipid species), and lysophosphatidylserine (LPS, 8 lipid species) were identified only at 12 hpi, and some lipid species of phosphatidylethanol (PEt, 11 and 8 lipid species), phosphatidylglycerol (PG, 17 and 3 lipid species), and triglyceride (TG, 31 and 23 lipid species) were identified at 12 and 24 hpi, respectively. Additionally, more lipid species of phosphatidylserine (PS) were identified at 36 dpi (12 lipid species) compared with 12 hpi (2 lipid species) or 24 hpi (2 lipid species) (**Figure 3C**). The lipid species with differential abundance were clustered at three infection stages, showing that the infection and control groups can be separated at each infection stage by hierarchical cluster analysis (**Supplementary Figure 6**). These results show that lipidomics can identify lipids with differential abundance and discriminate between the early and late stages of *T. canis* infection.

### Metabolic pathway alterations at different infection stages

**Figure 4:**
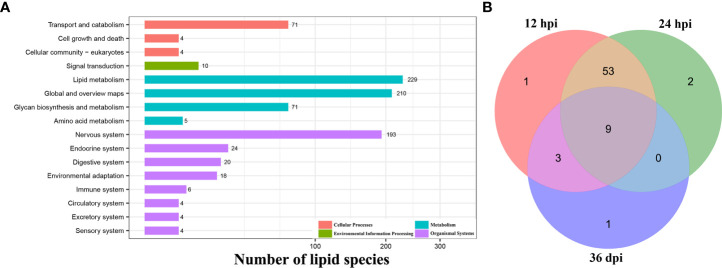
Functional enrichment analysis. **(A)** The KEGG pathway enrichment analysis of all identified lipid species at level 1 and level 2 classification. The X-axis represents the number of lipid species, and the Y-axis represents the KEGG pathway at level 2 classification. **(B)** Venn diagram shows the common and unique differential metabolic pathways among the three infection stages.

A total of 233 lipid species were enriched into 16 signaling pathways at level 2 classification by KEGG pathway enrichment analysis, and “lipid metabolism” in the item “Metabolism”, “nervous system” in the item “Organismal Systems”, “transport and catabolism” in the item “Cellular Processes”, and “signal transduction” in the item “Environmental Information Processing” were highly enriched. However, many of the identified lipid species do not have a clear enrichment or classification by KEGG analysis (**Figure 4A** and **Supplementary Table 3**).

Additionally, 69 differential metabolic pathways were identified after *T. canis* infection by KEGG analysis. At 12 hpi, eight differentially abundant lipid species were involved in 66 metabolic pathways, namely DG (16:0/18:1), PC (14:0/22:6), PC (35:4), PE (18:0/16:0), PE (44:11), LPA (18:0), TG (16:0/16:0/16:1), and LPC (22:5). At 24 hpi, four differentially abundant lipid species were involved in 64 metabolic pathways, namely Cer (d18:1/16:0), DG (16:0/18:1), PE (22:6/22:6), and TG (16:0/16:0/16:1). At 36 dpi, five differentially abundant lipid species were involved in 13 metabolic pathways, namely PC (14:0/14:0), PC (35:4), TG (18:1/18:2/20:4), LPC (22:4), and PS (18:0/18:1) (**Supplementary Table 4**).

At 12 and 24 hpi, some differentially abundant lipid species were significantly enriched in immune- or inflammation-related KEGG pathways, such as the B-cell receptor signaling pathway, the C-type lectin receptor signaling pathway, the NF-kappa B signaling pathway, and the Th1 and Th2 cell differentiation pathway. Of the 69 identified differential metabolic pathways, nine (13.0%) pathways, such as cholesterol metabolism, fat digestion and absorption, and vitamin digestion and absorption pathway, were identical at 12 hpi, 24 hpi, and 36 dpi (**Figure 4B**).

## Discussion

This is the first global analysis of liver lipidome in the context of *T. canis* infection of beagle dogs. LC-MS/MS profiling identified 173, 64, and 116 differentially abundant lipid species at 12 hpi, 24 hpi, and 36 dpi, respectively. The numbers of upregulated lipid species were markedly greater than those of the downregulated lipid species at the three infection stages. In a previous study, *T. canis* larvae were not detected in the liver of infected beagle puppies at 12 hpi, and when compared to the control puppies, no significant differences were observed in eosinophil counts or specific anti-*T. canis* IgG antibody in infected puppies at 12 hpi (Zheng et al., 2019). Additionally, fewer differential ions were identified in the serum at 12 hpi compared with later infection stages (Zheng et al., 2019). However, in the present study, *T. canis* infection significantly altered 12 lipid subclasses at 12 hpi, which was far more than the alterations detected at 24 hpi or 36 dpi. The increased level of hepatic lipid changes at this early stage of *T. canis* infection (12 hpi) might be an active response induced by the invading larvae.

At 12 hpi, the upregulation of LPG 2.28 times was the most significant alteration and included 11 upregulated LPG lipid species, suggesting that these lipids may be involved in the migration and development of *T. canis*. LPG is a member of lysophospholipids (LPLs), which serve as mediators *via* G-protein-coupled receptors (Makide et al., 2009). In mammalian tissues, LPG is a precursor for *de novo* synthesis of PG, which plays roles in phosphorylation of signaling molecules of extracellular signal-regulated kinase (ERK), increasing the concentration of intracellular Ca^2+^ and stimulating the chemotactic migration of natural killer cells (Park et al., 2007; Jo et al., 2008; Zhang et al., 2017). The increased intracellular Ca^2+^ by LPG may play a role in vascular smooth muscle cell elasticity and adhesion, and vascular dysfunction (Zhu et al., 2019). The involvement of LPG in *T. canis* interaction with and crossing the host blood vessels during the initial phase of infection warrants further investigation.

LPS, a member of LPLs, was upregulated 1.77 times at 12 hpi, including eight upregulated LPS lipid species. LPS is the deacylated form of phosphatidylserine (PS) and has a stimulatory effect on mast cell degranulation, enhancing histamine release from mast cells (Omi et al., 2021). LPS also has immune-modulatory functions, such as suppressing T lymphocyte proliferation and enhancing apoptotic cell engulfment by macrophages (Bellini and Bruni, 1993; Frasch et al., 2008). LPS from the blood fluke *schistosome* is a TLR2-activating molecule and can induce regulatory T cells, and monoacylated LPS can promote the development of IL-10-producing T cells (van der Kleij et al., 2002). The role of LPS in the pathogenesis of *T. canis* infection is unknown, and LPS was not identified in the non-protein ESP complement of *T. canis* (Wangchuk et al., 2020). We speculated that LPS may play a role in mitigating excessive inflammatory reactions of the hosts or attenuating the host immunity to establish an environment permissive for the parasite development; however, this remains to be investigated.

At 12 hpi, five LPC lipid species were identified with differential abundance, such as 7.55 times upregulation of LPC (16:0p). LPA is another member of LPLs that was upregulated 1.82 times at 12 hpi, including two upregulated LPG lipid species. LPA is produced from LPC by activation of platelets, neuronal cells, and adipocytes in humans and has high specificity to receptors coupled to G proteins, as well as promotes the proliferation of *Trypanosoma cruzi* (Chagas-Lima et al., 2019). Schistosomal-derived LPC can trigger M2 polarization of macrophages and activates human eosinophils, producing pro-inflammatory and immunoregulatory mediators (Assunção et al., 2017; Magalhães et al., 2018). The relevance of LPLs in immune modulation during early *T. canis* infection remains to be elucidated. Although most of the differential subclasses were upregulated, PEts were downregulated significantly at 12 hpi, including 11 downregulated PEt lipid species, such as downregulation 4.30 times of PEt (18:1/20:4) and 4.21 times of PEt (18:2/20:4). The role of PEts in the pathogenesis of *T. canis* infection merits investigation.

At 24 hpi where most *T. canis* larvae are present in the liver (Schnieder et al., 2011), the liver appears to partly regain homeostasis with only 64 lipid species with differential abundance and 2 altered lipid subclasses (Cer and TG). Ceramides (Cer) play roles in signal transduction in vital processes, such as apoptosis and cell differentiation (Takabe et al., 2008; Tanase et al., 2021). Cer was upregulated 1.25 times and 1.26 times, including one upregulated and five upregulated Cer lipid species at 12 and 24 hpi, respectively. Cer is also involved in inflammatory and metabolic pathways in hepatic steatosis and cardiovascular pathologies (Uchida and Park, 2021). The upregulation of Cer may be an adaptive mechanism used by *T. canis* to facilitate larval penetration through a dysfunctional vascular barrier and to promote its development *via* induction of apoptosis. Induction of apoptosis is a common mechanism used by several helminths to survive in the host (Zakeri, 2017). At 12 and 24 hpi, TG was upregulated 3.33 and 1.45 times, including 31 upregulated and 23 upregulated TG lipid species, respectively, without any downregulated TG lipid species. Some helminths hijack host lipids for their own growth and survival (Zinsou et al., 2020). For example, TGs are major precursors of lipids in *T. cruzi*, and blocking the supply of TGs disrupts the development of *T. cruzi* (Gazos-Lopes et al., 2017).

Previous studies have detected alterations in the transcriptome of the liver, lung, and bone marrow, even at 36 dpi where *T. canis* is settled in the intestine (Zou et al., 2020; Zheng et al., 2021a; Zheng et al., 2021b). In agreement with previous findings, *T. canis* also influenced the lipidome of the liver at 36 dpi, causing significant alterations in 116 lipid species and 4 lipid subclasses (SM, PMe, PS, and CerG3GNAc2). SM has been detected in helminths, although its biogenesis pathways are not well characterized (Bankov et al., 1998). A previous study showed that SM liposomes carrying *Leishmania* antigens induce strong Th2 immune response in mice (Biari et al., 2021), suggesting that SM can modulate the host immune response. PMe was also upregulated 1.79 times, including 11 upregulated PMe lipid species at 36 dpi, such as PMe (18:1/18:2) and PMe (18:0/20:4). The role of *T. canis*-related upregulation of SM and PMe in liver pathology and modulation of immune response merits further investigation.

To explore the interrelationship between differentially abundant lipid species induced by *T. canis* infection, we performed metabolic pathway analysis. Only 233 lipid species were enriched in signaling pathways, and 75.16% identified lipid species were not enriched by KEGG analysis. Some differentially abundant lipid species were enriched in immune- or inflammation-related KEGG pathway, such as Th1 and Th2 cell differentiation, the B-cell receptor signaling pathway, the C-type lectin receptor signaling pathway, and the NF-kappa B signaling pathway, suggesting that lipids, especially DG (16:0/18:1), play roles in regulating the immune or inflammatory responses of the host during *T. canis* infection. Nine differential metabolic pathways were commonly detected at all infection stages, such as the fat digestion and absorption, glycerolipid metabolism, and glycerophospholipid metabolic pathways, suggesting that these pathways may play crucial roles during *T. canis* infection. GP, a member of the glycerophospholipid metabolic pathway, is abundant in mammalian cell membranes, and the disturbance of its metabolism can lead to a range of diseases in humans and animals (Hermansson et al., 2011; van der Veen et al., 2017). The role of GP and other dysregulated metabolic pathways in the pathogenesis of *T. canis* infection warrants further investigation.

## Conclusion

Mass spectrometry-based lipidomics of the liver of beagle dogs infected by *T. canis* revealed significant alterations in the liver lipidome of the infected animals. The impact of *T. canis* on lipid homeostasis persisted even after the parasite settled in the small intestine. The lipidomic analysis also identified infection stage-specific lipid patterns and highlighted the potential involvement of many lipid species in mediating host–parasite interaction, particularly during the early stage of *T. canis* infection. Future studies that confirm the association between the altered lipid species and the mechanisms mediating the pathophysiology of *T. canis* infection may generate new opportunities for the discovery of possible targets for mitigating the clinical impact of hepatic toxocariasis.

## Data availability statement

The datasets presented in this study can be found in online repositories. The names of the repository/repositories and accession number(s) can be found in the article/**Supplementary Material**.

## Ethics statement

The animal study was reviewed and approved by the Animal Research Ethics Committee of Lanzhou Veterinary Institute, Chinese Academy of Agricultural Sciences (Approval No. 2018-015). The Beagle dogs used in this study were handled in accordance with good animal practice as defined by the relevant Animal Ethics Procedures and Guidelines of the People’s Republic of China.

## Author contributions

W-BZ, HME, and X-QZ conceived and designed the study and critically revised the manuscript. H-YL, YZ, and W-BZ performed the experiment, analyzed the lipidomics data, and drafted the manuscript. YX, LC, and S-CX helped in data analysis and manuscript revision. All authors contributed to the article and approved the submitted version.

## Funding

Project support was provided by the Science and Technology Innovation Program of Shanxi Agricultural University (Grant No. 2021BQ09), the Fund for Shanxi “1331 Project” (Grant No. 20211331-13), the Special Research Fund of Shanxi Agricultural University for High-level Talents (Grant No. 2021XG001), Yunnan Expert Workstation (Grant No. 202005AF150041), and Veterinary Public Health Innovation Team of Yunnan Province (Grant No. 202105AE160014). The funders had no role in study design, data collection and analysis, decision to publish, or preparation of the manuscript.

## Acknowledgments

We thank BGI-Shenzhen for technical assistance with the LC-MS/MS analysis.

## Conflict of interest

The authors declare that the research was conducted in the absence of any commercial or financial relationships that could be construed as a potential conflict of interest.

## Publisher’s note

All claims expressed in this article are solely those of the authors and do not necessarily represent those of their affiliated organizations, or those of the publisher, the editors and the reviewers. Any product that may be evaluated in this article, or claim that may be made by its manufacturer, is not guaranteed or endorsed by the publisher.

## Supplementary material

Supplementary Figure 1 The BPC (base peak chromatograms) overlapping spectrum of QC samples in positive ion mode (ESI^+^) **(A)** and negative ion mode (ESI^−^) **(B)**.

Supplementary Figure 2 The coefficient of variation (CV) and principal component analysis (PCA) of QC samples. **(A)** The CV plot of QC samples. The two lines perpendicular to the X-axis represent 20% and 30% CV reference lines, respectively, and the line parallel to the X-axis denotes 60% reference line. **(B)** The PCA score scatter plots of lipids in QC of infected **(I)** and control **(C)** samples. AG, A group; BG, B group; CG, C group.

Supplementary Figure 3 Total ion chromatography data show minor changes in the relative lipid abundance between the infected group **(I)** and control group **(C)** at 12 hpi **(A–D)**, 24 hpi **(E–H)**, and 36 dpi **(I–L)** in positive ion mode (ESI^+^) and negative ion mode (ESI^−^).

Supplementary Figure 4 The principal component analysis (PCA) score scatter plots of lipids in beagle dogs infected with 300 *Toxocara canis* eggs. (**A–C**) represent PCA score plots of the infected groups **(I)** and control groups **(C)** at 12 hpi, 24 hpi, and 36 dpi, respectively. AG, A group; BG, B group; CG, C group.

Supplementary Figure 5 The changes of lipid subclasses between the infected group **(I)** and control group **(C)** at 12 hpi, 24 hpi, and 36 dpi, respectively, by Student’s *t*-test using SPSS 19. **p* < 0.05, ***p* < 0.01, ****p* < 0.001.

Supplementary Figure 6 Heatmaps and hierarchical clustering of the lipid species with differential abundance between the infected groups **(I)** and control groups **(C)** at 12 hpi **(A)**, 24 hpi **(B)**, and 36 dpi **(C)**. The dendrograms at the top of the heatmaps represent the relationship between the different infected and control samples. The dendrogram at the left of the heatmaps represents the phylogenetic relationship between the lipid species.

## Abbreviations

LPLs: lysophospholipids; LPG: lysophosphatidylglycerol; LPS: lysophosphatidylserine; LPC: lysophosphatidylcholine; VLM: visceral larva migrans; OLM: ocular larva migrans; NT: neurotoxocariasis; ESPs: excretory–secretory products; FA: fatty acyls; GL: glycerolipids; GP: glycerophospholipids; PK: polyketides; PR: prenol lipids; SL: saccharolipids; SP: sphingolipids; ST: sterol lipids; SPF: specific pathogen free; QC: quality control; ACN: acetonitrile aqueous solution; FA: formic acid; AF: ammonium formate; MIT: maximum injection time; AGC: automatic gain control; CV: coefficient of variation; PCA: principal component analysis; PLS-DA: partial least-squares method—discriminant analysis; VIP: variable importance for projection; KEGG: Kyoto Encyclopedia of Genes and Genomes; BPC: base peak chromatograms; RSD: the relative standard deviation; PC: phosphatidylcholine; LPA: lysophosphatidic acid; PIP2: phosphatidylinositol 4,5-bisphosphate; Cer: ceramides; TG: triglyceride; SM: sphingomyelin; PMe: phosphatidylmethanol; PS: phosphatidylserine; DG: diglyceride; PE: phosphatidylethanolamine; CerG2: diglycosylceramide; PEt: phosphatidylethanol; PG: phosphatidylglycerol; ERK: extracellular signal-regulated kinase; GC: gas chromatography; S1P: sphingosine1-phosphate.

## Glossary

**Table:**

LPLs	lysophospholipids
LPG	lysophosphatidylglycerol
LPS	lysophosphatidylserine
LPC	lysophosphatidylcholine
VLM	visceral larva migrans
OLM	ocular larva migrans
NT	neurotoxocariasis
ESPs	excretory–secretory products
FA	fatty acyls
GL	glycerolipids
GP	glycerophospholipids
PK	polyketides
PR	prenol lipids
SL	saccharolipids
SP	sphingolipids
ST	sterol lipids
SPF	specific pathogen free
QC	quality control
ACN	acetonitrile aqueous solution
FA	formic acid
AF	ammonium formate
MIT	maximum injection time
AGC	automatic gain control
CV	coefficient of variation
PCA	principal component analysis
PLS-DA	partial least-squares method—discriminant analysis
VIP	variable importance for projection
KEGG	Kyoto Encyclopedia of Genes and Genomes
BPC	base peak chromatograms
RSD	the relative standard deviation
PC	phosphatidylcholine
LPA	lysophosphatidic acid
PIP2	phosphatidylinositol 4,5-bisphosphate
Cer	ceramides
TG	triglyceride
SM	sphingomyelin
PMe	phosphatidylmethanol
PS	phosphatidylserine
DG	diglyceride
PE	phosphatidylethanolamine
CerG2	diglycosylceramide
PEt	phosphatidylethanol
PG	phosphatidylglycerol
ERK	extracellular signal - regulated kinase
GC	gas chromatography
S1P	sphingosine1-phosphate.
