# Supplementary figures and images for: Lipidomic changes in the liver of beagle dogs associated with Toxocara canis infection
Source: Front Cell Infect Microbiol. 2022 Sep 13;12:890589. doi: 10.3389/fcimb.2022.890589 (PMC9514057; doi:10.3389/fcimb.2022.890589)

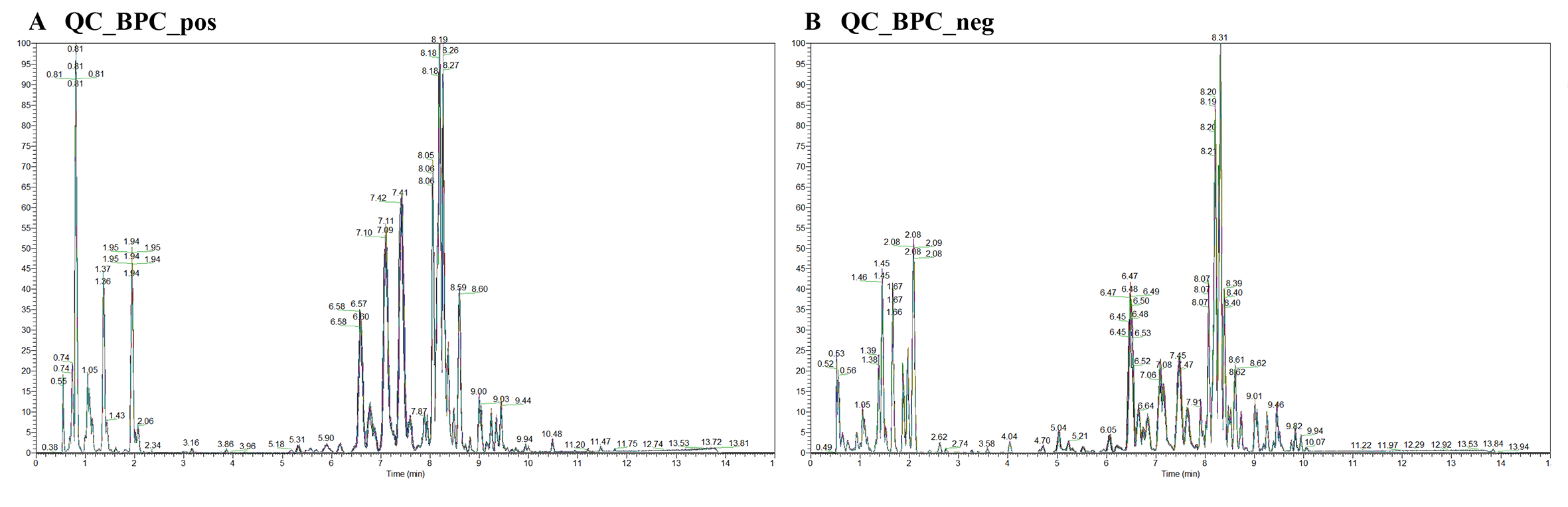

Supplement: Supplementary Figure 1 — The BPC (base peak chromatograms) overlapping spectrum of QC samples in positive ion mode (ESI+) (A) and negative ion mode (ESI−) (B). [file Image_1.tif]

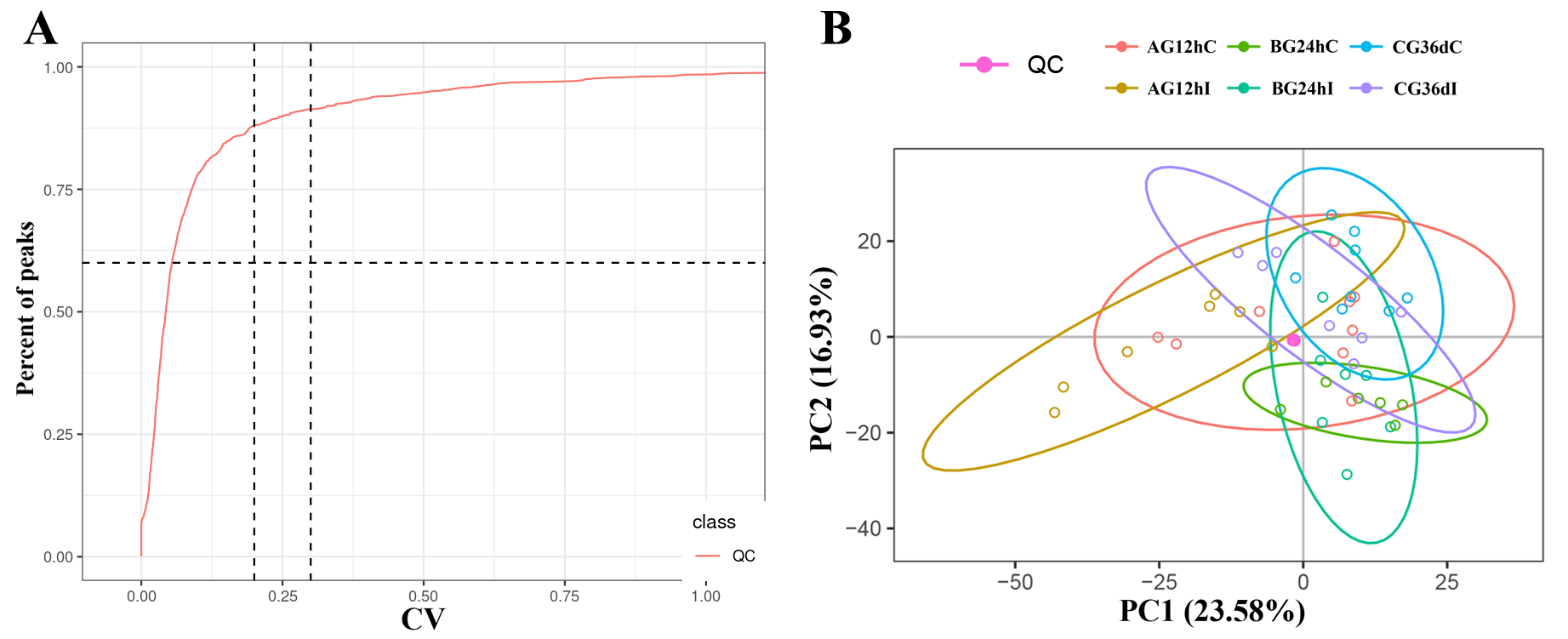

Supplement: Supplementary Figure 2 — The coefficient of variation (CV) and principal component analysis (PCA) of QC samples. (A) The CV plot of QC samples. The two lines perpendicular to the X-axis represent 20% and 30% CV reference lines, respectively, and the line parallel to the X-axis denotes 60% reference line. (B) The PCA score scatter plots of lipids in QC of infected (I) and control (C) samples. AG, A group; BG, B group; CG, C group. [file Image_2.tif]

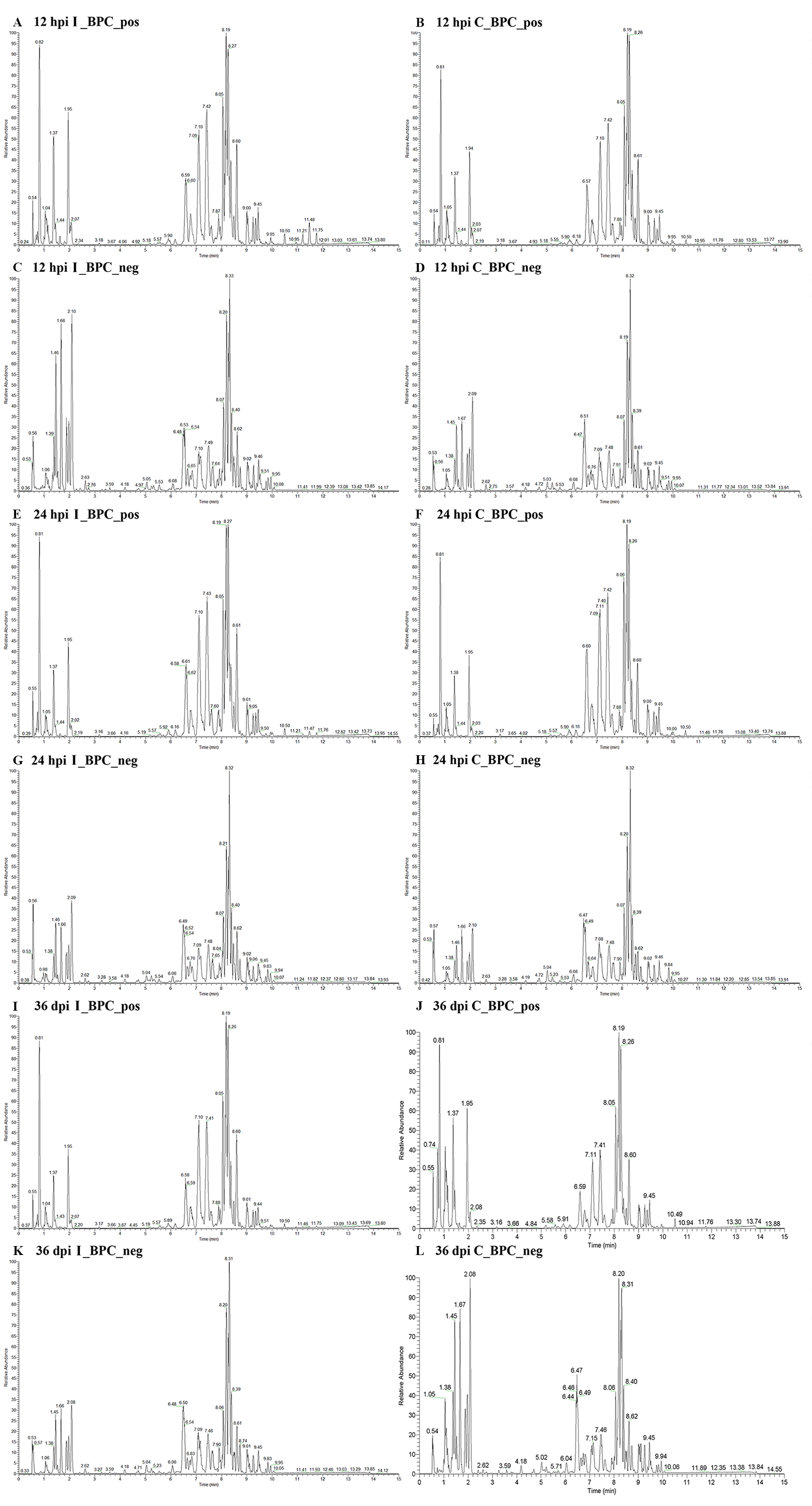

Supplement: Supplementary Figure 3 — Total ion chromatography data show minor changes in the relative lipid abundance between the infected group (I) and control group (C) at 12 hpi (A–D), 24 hpi (E–H), and 36 dpi (I–L) in positive ion mode (ESI+) and negative ion mode (ESI−). [file Image_3.tif]

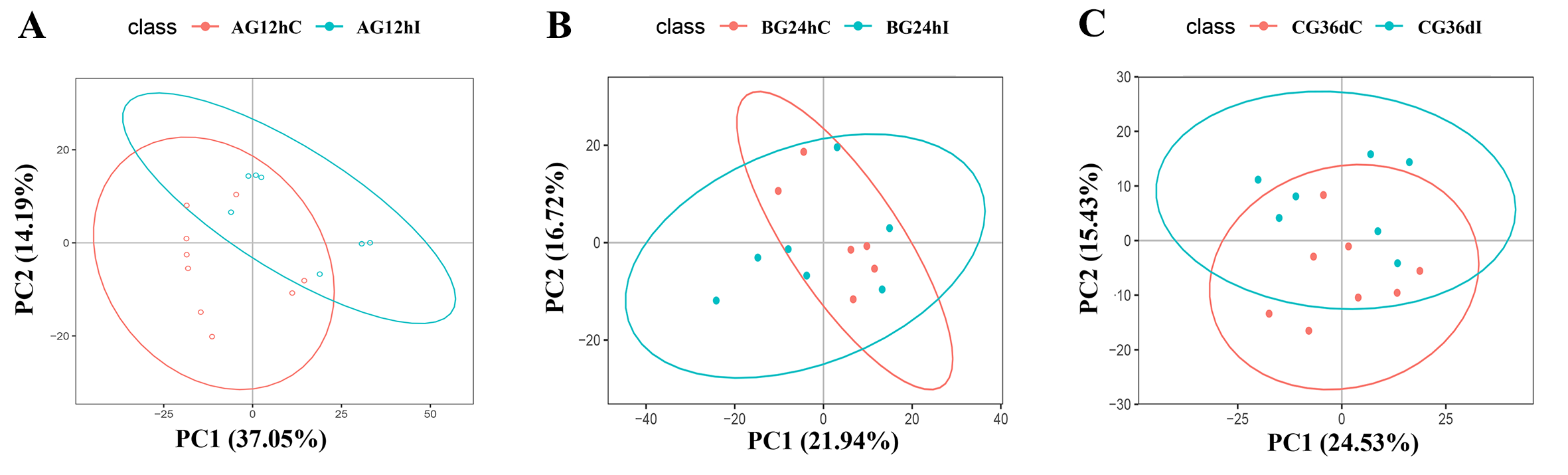

Supplement: Supplementary Figure 4 — The principal component analysis (PCA) score scatter plots of lipids in beagle dogs infected with 300 Toxocara canis eggs. (A–C) represent PCA score plots of the infected groups (I) and control groups (C) at 12 hpi, 24 hpi, and 36 dpi, respectively. AG, A group; BG, B group; CG, C group. [file Image_4.tif]

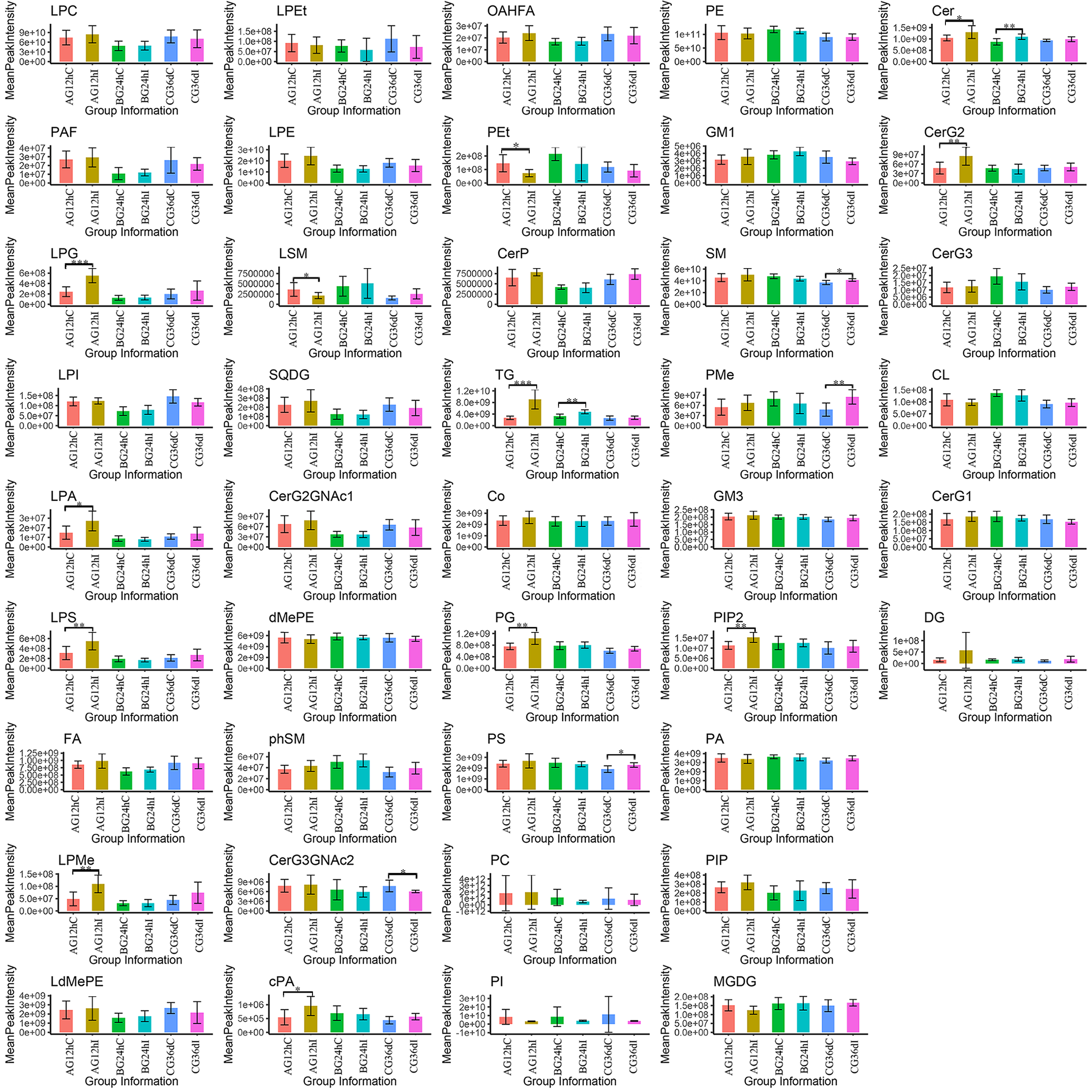

Supplement: Supplementary Figure 5 — The changes of lipid subclasses between the infected group (I) and control group (C) at 12 hpi, 24 hpi, and 36 dpi, respectively, by Student’s t-test using SPSS 19. *p < 0.05, **p < 0.01, ***p < 0.001. [file Image_5.tif]

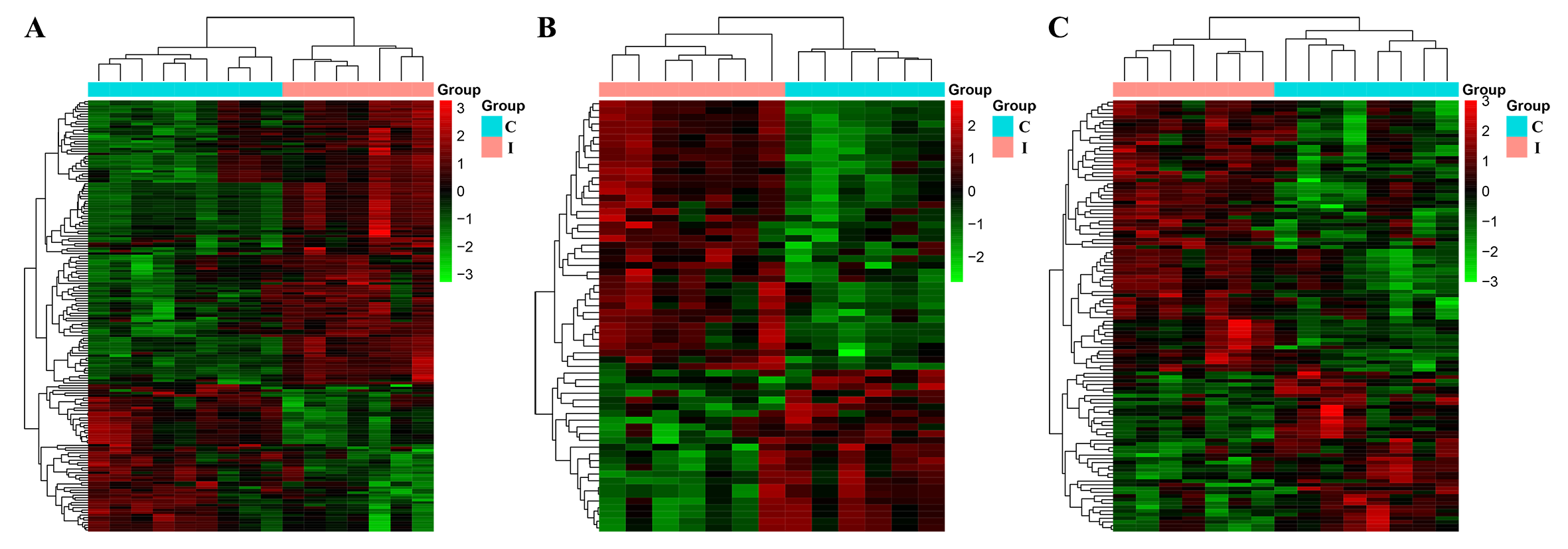

Supplement: Supplementary Figure 6 — Heatmaps and hierarchical clustering of the lipid species with differential abundance between the infected groups (I) and control groups (C) at 12 hpi (A), 24 hpi (B), and 36 dpi (C). The dendrograms at the top of the heatmaps represent the relationship between the different infected and control samples. The dendrogram at the left of the heatmaps represents the phylogenetic relationship between the lipid species. [file Image_6.tif]
